# Guideline-Adherent Physiotherapy for Patients With Hip and Knee Osteoarthritis in Germany: Protocol for an Implementation Research Project Using the Theoretical Domains Framework and the Behavior Change Wheel

**DOI:** 10.2196/47834

**Published:** 2023-11-16

**Authors:** Carolin Bahns, Bettina Scheffler, Christian Kopkow

**Affiliations:** 1 Department of Therapy Science I Brandenburg University of Technology Cottbus-Senftenberg Senftenberg Germany

**Keywords:** osteoarthritis, physiotherapy, guideline, implementation, clinical practice

## Abstract

**Background:**

Hip and knee osteoarthritis is common and leads to pain, stiffness, and disability. Clinical practice guidelines provide recommendations based on the best available evidence to assist health care professionals and patients in clinical decision-making. However, several studies have reported a gap between guideline recommendations and clinical practice in physiotherapy. Improved implementation strategies and the removal of existing barriers may facilitate the transfer of evidence into clinical practice and contribute to optimized quality of care.

**Objective:**

This paper presents the protocol for a study that aims to describe the current physiotherapy practice in patients with hip and knee osteoarthritis and to investigate physiotherapists’ adherence to clinical practice guidelines, to identify and specify barriers to and facilitators of guideline use and implementation, and to develop and pilot test a theory-based tailored implementation intervention aiming to increase guideline use in osteoarthritis care.

**Methods:**

The research project is divided into 4 parts. During the first part, we will conduct a nationwide web-based survey among German physiotherapists to evaluate the current management of hip and knee osteoarthritis and to evaluate whether treatment aligns with guideline recommendations. Subsequently, semistructured interviews will be conducted to specify barriers to and facilitators of guideline use and implementation among physiotherapists (part 2). On the basis of these findings, in part 3, we will develop a theory-driven implementation intervention based on the Theoretical Domains Framework and the Behavior Change Wheel, which will be evaluated in a controlled pilot study in terms of effectiveness, feasibility, and acceptability (part 4).

**Results:**

Data collection of the web-based survey among German physiotherapists (part 1) was completed in December 2021. The semistructured interviews (part 2) were conducted between January and September 2023. Recruitment of physiotherapy practices to participate in the development of the implementation intervention is expected to start in January 2024.

**Conclusions:**

This research project aims to develop a theory-driven implementation intervention to facilitate the transfer of evidence from hip and knee osteoarthritis guidelines in physiotherapy practice. We hypothesize that successful implementation will lead to increased guideline adherence in physiotherapists, which in turn will improve the quality of care. The results from our project will provide valuable knowledge concerning the development process and effectiveness of tailored implementation interventions.

**International Registered Report Identifier (IRRID):**

DERR1-10.2196/47834

## Introduction

Osteoarthritis is the most common chronic joint disease and is mainly present in the lower extremity joints such as hips and knees [1]. In Germany, the prevalence of outpatient diagnosed hip and knee osteoarthritis in older patients is 21.8%, whereas osteoarthritis is more common in women than in men and its prevalence increases with age [2]. Osteoarthritis is characterized by inflammation and degenerative changes in the joints, causing pain, stiffness, functional disability, and reduced quality of life [3]. In addition to its effects on individuals, osteoarthritis is associated with an enormous burden on the health care system. The average annual costs per patient living with osteoarthritis in the lower extremities worldwide were estimated to be €11,100 (US $11,722) [4]. In Germany, the direct costs for osteoarthritis (*International Classification of Diseases, 10th Revision*: M15-M19) amounted to €7.62 (US $8.05) billion in 2008. Data on indirect costs are not available, but with 114,975 cases of work incapacity owing to knee osteoarthritis and 44,637 cases owing to hip osteoarthritis (2011), an enormous burden on the national economy can be assumed [5].

There are numerous treatment options available including conservative (eg, physiotherapy) and operative methods to manage hip and knee osteoarthritis, which vary with respect to efficacy and costs. To facilitate the implementation of research findings in clinical practice, clinical practice guidelines (CPGs) summarizing study results have been developed. CPGs are described as “statements that include recommendations intended to optimize patient care that are informed by a systematic review of evidence and an assessment of the benefits and harms of alternative care options” [6].

In recent CPGs [7-9], conservative nonpharmacological methods, particularly education and therapeutic exercise (and weight loss if overweight or obese), are widely recommended and are seen as first-line treatment for patients with hip and knee osteoarthritis. Other interventions (eg, pain medication or gait aids) can be used to relieve pain and to support first-line treatment. Joint replacement surgery is usually considered when osteoarthritis symptoms become intolerable and significantly affect quality of life, and other nonsurgical therapies have been ineffective or unsuitable [9].

Physiotherapeutic interventions play a key role in the conservative management of hip and knee osteoarthritis and have been shown to be effective in improving pain, quality of life, and physical function [10]. On the basis of the German health insurance data, Postler et al [2] identified that approximately 63.4% of patients (aged ≥60 y) diagnosed with hip or knee osteoarthritis receive pain medication (44.1% of which are nonsteroidal anti-inflammatory drugs), whereas physiotherapy is prescribed only to 43.1% of the patients, with decreasing numbers with increasing age.

However, deficits in osteoarthritis management are not only evident in physicians’ prescribing behavior. Recent studies involving physiotherapists from Italy [11] and Belgium [12] indicated a gap between clinical practice and research findings, as treatment of hip and knee osteoarthritis often is not in line with current guideline recommendations. Instead of providing first-line treatments, many physiotherapists include interventions that do not contribute to high-quality care. This is consistent with findings from a systematic review in which Zadro et al [13] found that the median percentage of physiotherapists who chose treatments recommended in CPGs (eg, advice to stay active, aerobic, and strengthening exercises) was 58%, whereas 98% of the physiotherapists provided treatments with low or conflicting evidence in the management of knee osteoarthritis (eg, exercise that is neither aerobic nor strengthening). With regard to the nonpharmacological treatment of people with osteoarthritis, Hagen et al [14] noted that there is substantial room for improvement in the care of individual patients.

The limited use of evidence in clinical practice is a well-known problem, and the reasons for not using CPGs are manifold. Important barriers to the implementation of CPGs in physiotherapy mentioned in the literature are, for example, lack of time, lack of generalizability of research findings to individual patients, conflicting patient preferences, and lack of support in the workplace [15-19]. In addition, physiotherapists are often unaware of current CPGs [20,21]. Owing to the characteristics of the German health care system and the education of most physiotherapists in Germany at vocational schools instead of at institutions of higher education, it can be assumed that there are further barriers specific to the German context.

Publication and dissemination of guidelines alone is not sufficient to change clinical practice. It requires organizational change and behavioral change in individual health care professionals toward their use of guideline recommendations. Therefore, CPGs need to be accompanied by evidence-based implementation strategies [17]. There is evidence that tailored implementation programs targeting specific barriers and facilitators are more likely to improve professional practice than nontailored interventions or passive guideline dissemination [22,23]. It is recommended to use theories and frameworks to guide the implementation process and facilitate the identification of determining factors in health care professionals’ behavior [24]. Moreover, using theories allows for a better understanding of the generalizability and replicability of implementation interventions [25].

A promising approach to developing a tailored, theory-driven implementation intervention might be the involvement of the Theoretical Domains Framework (TDF) and the Behavior Change Wheel (BCW). As one of the most commonly used theories in health care settings [26], the TDF synthesizes different theories of behavior change into one framework and is commonly used to explore factors that influence clinical behavior and guideline adherence. The 14 domains of the TDF include knowledge; skills; social or professional role and identity; beliefs about capabilities; optimism; beliefs about consequences; reinforcement; intentions; goals; memory, attention, and decision processes; environmental context and resources; social influences; emotion; and behavioral regulation [27]. The BCW is a synthesis of 19 theoretical frameworks of behavior change and provides a systematic approach to developing a tailored guideline implementation intervention. The barriers and facilitators identified through the TDF can be used to expand the Capability, Opportunity, Motivation–Behavior components (COM-B), the core of the BCW, which indicates that behavior change occurs when there is a change in an individual’s capability, opportunity, and motivation [28].

To date, little is known regarding the current physiotherapy practice for hip and knee osteoarthritis in Germany and whether its management is in line with guideline recommendations. We hypothesize that guideline adherence among physiotherapists is low, as in Germany, an academic level of physiotherapy education is not mandatory and only about 3% of the physiotherapists have graduated from higher educational institutions [29]. Most physiotherapists in Germany are trained at vocational schools where skills and knowledge regarding the implementation of guidelines and evidence-based practice are not an integral part of the curriculum. Given the burden of the disease and the high costs associated with osteoarthritis, as well as the importance of physiotherapy interventions in the treatment of hip and knee osteoarthritis, it is important to address this lack of research. Developing a tailored implementation intervention can increase evidence uptake in clinical practice and thus, improve quality of health care.

The objectives of the research project are (1) to describe the current physiotherapy practice in patients with hip and knee osteoarthritis in Germany and to investigate physiotherapists’ adherence to CPG recommendations; (2) to identify and specify barriers to and facilitators of guideline use and implementation; and (3) to develop and (4) pilot test a theory-based tailored implementation intervention that aims to increase guideline use in the physiotherapy care of patients with hip and knee osteoarthritis in the German context.

## Methods

### Overview

**Figure 1:**
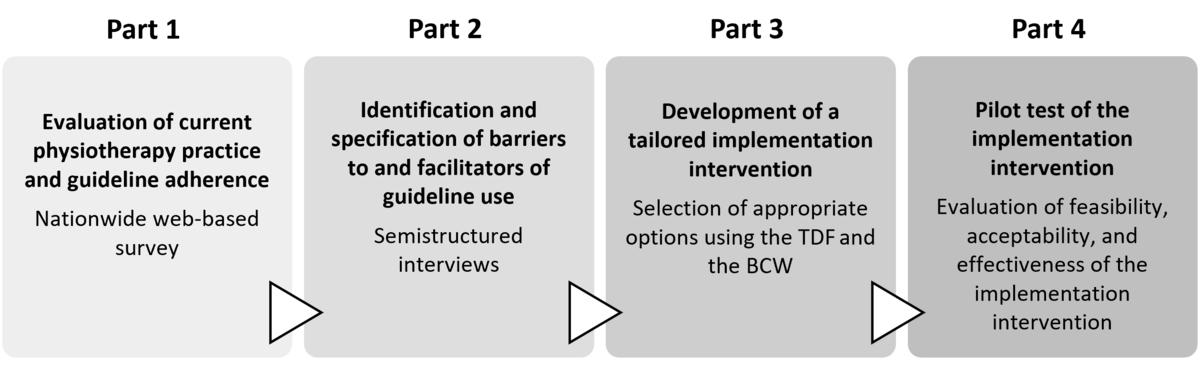
Flowchart of study parts. BCW: Behavior Change Wheel; TDF: Theoretical Domains Framework.

The study was divided into 4 parts (Figure 1). In the first part, we will evaluate the current physiotherapy practice regarding the management of patients with hip and knee osteoarthritis. Subsequently, we will identify and specify barriers to and facilitators of guideline use among physiotherapists using a qualitative approach. On the basis of these findings, in part 3, we will develop a tailored implementation intervention to increase guideline use in clinical practice, which will be evaluated in a pilot study in part 4.

The evaluation and implementation of guideline-adherent care will be based on the German guidelines for hip and knee osteoarthritis [30,31]. These guidelines are aimed specifically at physicians working in musculoskeletal care and serve as information for general practitioners, physiotherapists, and occupational therapists, for example.

### Part 1: Evaluation of Current Physiotherapy Practice and Guideline Adherence

A nationwide web-based survey among physiotherapists will be conducted to evaluate the current management of patients with hip and knee osteoarthritis and to explore adherence from physiotherapists to guideline recommendations. The reporting of the study will follow the Strengthening the Reporting of Observational Studies in Epidemiology statement [32] and the Consensus-Based Checklist for Reporting of Survey Studies [33].

A self-administered questionnaire will be developed based on the recommendations regarding conservative nonpharmacological management listed in the German CPGs for hip and knee osteoarthritis [30,31]. The questionnaire will be divided into three sections to assess (1) the demographic and working characteristics of the participants, (2) the current physiotherapy practice in patients with hip and knee osteoarthritis, and (3) perceived barriers to the implementation and clinical use of guidelines. We will use a mix of multiple choice questions and yes or no questions. To evaluate the current clinical practice, study participants will be asked to rate different treatment modalities for osteoarthritis on a 4-point Likert-type scale (1=never, 2=sometimes, 3=mostly, and 4=always). Specific barriers and facilitators regarding the German guidelines for hip and knee osteoarthritis will be assessed using the translated and cultural adapted version of “The Barriers and Facilitators Assessment Instrument” [34]. For participants who reject using CPGs in clinical practice, reasons against guideline use will be explored based on barriers identified from literature. Adaptive questioning will be used to minimize the response burden and to reduce complexity of the questions. The web-based survey will be conducted using LimeSurvey, a free and open source web-based tool for research projects. The survey will be accessible without restrictions (password or registration) via an internet link to the LimeSurvey platform with no incentives offered. Before initiating the survey, the study participants have to read and accept written informed consent and are required to verify self-reported eligibility criteria. Participation will be voluntary and anonymous. To improve the quality and understanding of the questionnaire, a pretest of the web-based survey with 10 physiotherapists representative of the physiotherapists targeted with the survey will be conducted.

We will include physiotherapists mainly working in Germany who treat patients with hip or knee osteoarthritis in clinical practice and who are able to read and speak the German language. Only physiotherapists with a professional degree will be eligible; therefore, students will be excluded. The recruitment of participants will be initiated through announcements and calls on different physiotherapeutic networks, social media, and personal contacts of the study team. According to the snowball sampling approach, study participants will be invited to further distribute the participation invitation. As this study has an explorative character, no sample size calculation will be performed. There are approximately 203,000 physiotherapists working in Germany [29], but the number of physiotherapists involved in osteoarthritis care is unclear. We will target a total sample size of 1000 participants to allow regression analyses with subgroups of sufficient sample size. As survey participation is anonymous and different recruitment strategies will be used to maximize the response rate, there is a risk of duplicate responses. Therefore, we will systematically review all the responses to identify any surveys with matching demographics and answers.

Data analysis will be performed using the R software (version 4.0.3; R Foundation for Statistical Computing). Participants’ characteristics and current clinical practice will be analyzed using descriptive statistics. Guideline adherence will be defined as accordance between recommendations retrieved from the German guidelines for knee and hip osteoarthritis [30,31] and the therapists’ treatment choices. As there is no standardized method to evaluate guideline adherence, we will use 2 different approaches that have been recently used in previous studies. In the first approach, guideline adherence will be determined using a points system. The participant’s treatment choices will be compared with the current guideline recommendations, and in case of adherent behavior, each will be scored with one point. The benchmarks for good adherence will be set at ≥80% of the maximally achievable points [20]. Assessing guideline adherence on the basis of the methods of Battista et al [11], participants will be considered as *delivering*, *partially delivering*, and *nondelivering* depending on their chosen treatment. For example, a participant will be classified *delivering* if all recommended interventions have been chosen without considering nonrecommended treatments. To assess possible determinants of guideline adherence, regression analyses will be conducted. The potential determinants will be selected based on the literature. The level of statistical significance will be set at *P*<.05.

### Part 2: Identification and Specification of Barriers to and Facilitators of Guideline Use

A qualitative approach including semistructured interviews will be used to identify and specify barriers and facilitators to guideline use in the management of hip and knee osteoarthritis specifically for the German context. The reporting of this study will be in accordance with the Consolidated Criteria for Reporting Qualitative Research [35].

Purposive sampling will be used to gain a wide range of information. We aim to recruit a diverse sample of physiotherapists based on age, gender, work experience, and professional education level. Only physiotherapists mainly working in Germany who treat patients with hip or knee osteoarthritis in clinical practice and who are able to read and speak the German language will be included. The invitation to participate in one-to-one semistructured interviews will be distributed through announcements and calls on different physiotherapeutic networks, social media, and personal contacts of the study team. Recruitment will continue until data saturation has been reached (a minimum of 10 participants). Data saturation will be defined as the point when 3 consecutive interviews had been conducted with no additional information on barriers and facilitators emerging [36].

An interview guide will be developed based on the TDF [27]. Questions will be informed by key findings from the previous survey and information on barriers and facilitators to guideline use identified through literature. The guide will explore the domains of the TDF to understand the factors that influence guideline use in clinical practice in the management of hip or knee osteoarthritis. The interview guide will be pilot tested with 2 physiotherapists before use. Feedback on comprehension and content will subsequently be incorporated into the interview guide after discussion with the study team.

The interviews are anticipated to last approximately 45 to 60 minutes and will be conducted via telephone or video call. Before entering the study, the participants will receive a written informed consent. Interviews will be audio-recorded and transcribed verbatim. Data collection and storage will be done pseudonymously. To give participants the opportunity to review the transcript and to make any modifications, the transcripts will be sent to the participants for member checking.

Thematic analysis following the 6 stages from Braun and Clarke [37] will be used to analyze the interviews. The first transcript from the semistructured interview will be pilot coded independently by 2 researchers to develop a coding strategy. The remaining interviews will be analyzed iteratively by the first author who will discuss any difficulties with a second researcher. Finally, the barriers and facilitators will be mapped onto relevant domains from the TDF. Data will be analyzed using MAXQDA (version 22; VERBI GmbH) software.

### Part 3: Development of a Tailored Implementation Intervention

#### Overview

In this step, 6 outpatient physiotherapy practices will be recruited, 3 of which will be randomly selected to participate in the development process of the tailored intervention. In these physiotherapy practices, the intervention will be implemented (study part 4), whereas the remaining practices will serve as a control group. We will include physiotherapy practices in the region of Brandenburg and Saxony (Germany) with >5 physiotherapists who treat patients with hip or knee osteoarthritis in clinical practice.

**Figure 2:**
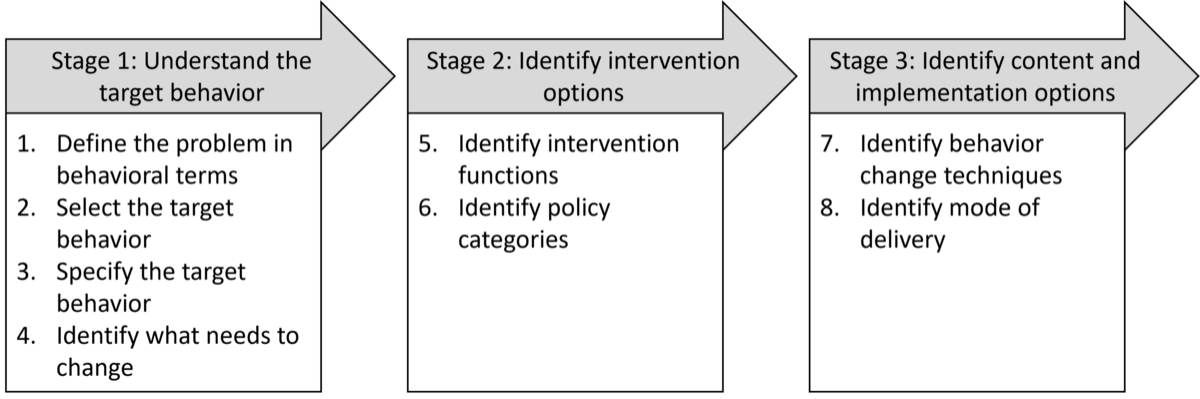
Stages and steps involved in the development of an intervention using the Behavior Change Wheel.

The development of the implementation intervention will be guided by the BCW and follows 3 stages, as outlined by Michie et al [28]: understand the target behavior, identify intervention options, and identify content and implementation options. These can be further divided into key substeps as illustrated in Figure 2 [28].

#### Stage 1: Understand the Target Behavior

The key barriers identified from the semistructured interviews (part 2) will be used to define the problem in behavioral terms and to specify the behavior and target population (step 1). From a list of all potential candidate behaviors, the study team will choose 1 or 2 core behaviors that appear to be promising in increasing guideline adherence in physiotherapists (step 2). The target behavior will be selected considering each potential impact, likelihood of change, spillover effect, and ease of measurement. After specifying the target behavior in detail and its context (who, what, when, where, how often, and with whom; step 3), focus groups will be conducted to identify what needs to change in the individual or the environment to achieve the desired behavior change (step 4).

The reporting of the focus groups will be guided by the Consolidated Criteria for Reporting Qualitative Research checklist [35]. One to 2 focus groups will be conducted at each participating physiotherapy practice. The focus groups will take place at the participants’ worksite and will be moderated by 2 members of the research team. Mixed groups of 6 to 10 participants recruited from each physiotherapy practice representing different stakeholders (eg, physiotherapists, patients, management, administration, and other health professionals) will be invited to participate to promote diversity and to encourage a wide range of information on what needs to change regarding the target behavior. All participants will receive and approve a written informed consent before initiating the study. The topic guide will be developed using the TDF. The standardized guide will be used across all focus groups with some flexibility through open questions to allow further discussion between participants. Before the focus groups, basic demographics (eg, age, gender, job type, and working hours) will be collected from each participant. The focus group discussions will be audio-recorded while simultaneously handwritten field notes capturing relevant nonverbal responses or dynamics of the group will be documented. Data from the focus groups will be transcribed verbatim and will be analyzed following the same process used for the semistructured interviews described in the *Part 2: Identification and Specification of Barriers to and Facilitators of Guideline Use* section.

#### Stage 2: Identify Intervention Options

On the basis of the findings from the focus groups, relevant intervention functions will be identified using a matrix that systematically maps TDF domains to intervention functions (eg, education, training, persuasion; step 5). As changes at the policy level will be beyond the scope and time constraints of our project, and intervention programs targeting individual level and local barriers have been shown to be effective [38], policy categories (eg, regulation, communication or marketing, and service provision) provided by the BCW guidance will not be considered in this study (step 6). To select the most appropriate and effective intervention functions for our implementation intervention, the affordability, practicability, effectiveness and cost effectiveness, acceptability, side effects or safety, and equity criteria will be used [28].

#### Stage 3: Identify Content and Implementation Options

On the basis of the intervention functions selected in step 5, behavior change techniques (BCTs) will be identified (step 7). We will use BCT taxonomy (version 1; BCTTV1) [39] to generate a list of BCTs mapped to our intervention functions. Regarding the affordability, practicability, effectiveness and cost effectiveness, acceptability, side effects or safety, and equity criteria, the research team will work in close collaboration with the stakeholders from the participating physiotherapy practices and discuss the included BCTs as well as possible modes of delivery for the intervention (step 8)*.* Those that are likely to be the most effective and feasible to increase guideline adherence will be selected. Reporting of the intervention developed will be consistent with the recommendations for specifying and reporting implementation strategies in research studies proposed by Proctor et al [40] and the Template for Intervention Description and Replication checklist [41].

### Part 4: Pilot Test of the Implementation Intervention

A controlled pilot study will be conducted to evaluate the effectiveness, feasibility, and acceptability of the tailored intervention implementation. The pilot study will simultaneously test a clinical intervention and an implementation intervention or strategy and thus will be a hybrid study [42]. The Standards for Reporting Implementation Studies statement [43] and the CONSORT (Consolidated Standards of Reporting Trials) statement [44] for pilot and feasibility trials will be used to guide the reporting of study methods and results.

The previously designed intervention will be implemented and evaluated at the 3 physiotherapy practices recruited in part 3 that participated in the development process, whereas the other 3 recruited practices will serve as the control group.

All physiotherapists involved in osteoarthritis management will be invited to participate. Patients will be eligible if they (1) are aged ≥18 years, (2) are primarily diagnosed with hip or knee osteoarthritis, and (3) have no restraint to receive physiotherapy. Participation will be voluntary, and informed consent will be obtained before the recruitment of any participant into the study.

To explore effectiveness at the patient level, the assessment of primary patient-relevant outcomes will follow the Outcome Measures in Rheumatology-Osteoarthritis Research Society International core domain set for clinical trials in hip and knee osteoarthritis and will include 5 domains: pain, physical function, quality of life, patient’s global assessment of the target joint, and adverse events [45]. Patient outcomes will be assessed at baseline, at the end of physiotherapy treatment, and after 6 months. Physiotherapists’ adherence to the osteoarthritis guidelines will be evaluated using a documentation questionnaire after each physiotherapy session. The implementation process will be evaluated using the Acceptability of Intervention Measure, Intervention Appropriateness Measure, and the Feasibility of Intervention Measure [46,47]. Additional outcomes will be the recruitment and retention rate [48]. Results from the pilot study will be used to inform sample size calculation for a subsequent study.

### Patient and Public Involvement Statement

Patients and the public were not directly involved in the design or conduct, reporting, or dissemination plans of this research.

### Ethical Considerations

Ethics approval for each part of the study will be sought from the ethics committee of the Brandenburg University of Technology Cottbus-Senftenberg, Germany. Permission for the nationwide web-based survey (study part 1) and the semistructured interviews (study part 2) has already been obtained from the ethics committee of the Brandenburg University of Technology Cottbus-Senftenberg, Germany (EK2021-10, EK2022-15). Throughout the research project, the principles established in the Declaration of Helsinki [49] will be strictly followed. Participants who are eligible for any part of the study will be informed about the respective objectives, content, and procedures of the study. Written informed consent will be obtained from all patients before enrollment. Participation will be voluntary, and participants are free to withdraw from the study at any moment.

The findings from this research project will be disseminated through open-access publications in international peer-reviewed journals and presentations at national and international scientific meetings. In addition, we will use social media (eg, X, formerly known as Twitter) to spread our findings to the public.

## Results

In December 2021, the survey among German physiotherapists was completed, and results on the management of patients with hip and knee osteoarthritis and guideline adherence were published [50] (part 1 of the research project). Ethics approval for the second part of the research project was obtained in August 2022, and the semistructured interviews were conducted between January and September 2023. Recruitment of physiotherapy practices to participate in the development of the implementation intervention is expected to start in January 2024.

## Discussion

### Overview

The described research project aims to evaluate and improve the implementation of hip and knee osteoarthritis guidelines in physiotherapy in Germany, which to our knowledge has not been performed to date. Considering the burden of disease and the importance of physiotherapy interventions in the management of osteoarthritis, as well as findings from other countries indicating gaps between clinical practice and research findings [11,12], it seems important to address this lack of research.

The implementation of research findings into clinical practice is considered relevant for improving quality of care but is often hindered by various barriers [51]. Although guidelines are considered helpful in translating research findings into clinical practice by providing recommendations that can be used by health care professionals, patients, policy makers, and other stakeholders, many guidelines include no or a variety of guideline implementation tools for different stakeholders [52]. There are few studies on how to choose implementation strategies that address guideline-specific barriers, but education for clinicians or patients and printed materials were the most commonly used strategies for translating guidelines into practice [53]. Holm et al [54] reported that the limited use of CPGs may be because of insufficient knowledge of current evidence or that clinicians and patients may find it difficult to break old habits and abandon existing low-value treatment and also because the increasing number of frameworks and checklists for guideline implementation makes implementation strategies too comprehensive and complicated.

For the management of people with osteoarthritis, pragmatic approaches to implementing osteoarthritis guidelines exist. For example, Good Life with osteoArthritis in Denmark (GLA:D) or Active with osteoArthritis (ActiveA) have been implemented in several countries [54]. GLA:D, which is currently being implemented in Germany, and ActiveA are both effective in reducing pain intensity and increasing quality of life, physical activity, or physical function [10,55-58]. Østerås et al [59] showed that a structured implementation of ActiveA in primary care resulted in a higher quality of osteoarthritis care as compared with usual care. However, it is unclear if such approaches can be implemented as standard care in the German health care system, as GLA:D does not fit into the German catalog of remedies, which defines the number and type of physiotherapeutic treatments, and is therefore only available based on special contracts with health insurance companies or must be paid for privately by patients. Furthermore, not all patients can (eg, owing to limited access to physiotherapy practices) or want to (eg, because they do not want to train in a group) participate in GLA:D. Therefore, it is necessary to evaluate how to increase guideline use in osteoarthritis care and thus improve the quality of care in addition to the ongoing implementation of GLA:D in Germany. With regard to physiotherapists in Germany, it was recently shown that the implementation of evidence-based practice in clinical practice is insufficient [60]. In a recent nationwide survey on physiotherapy management of low back pain, less than one-third of the participating physiotherapists reported being aware of the National Disease Management Guideline for “non-specific low back pain” or have dealt with its recommendations [20]. Similar results were shown by another survey, in which only 47% of the responding physiotherapists reported being aware of the German guidelines on stroke rehabilitation [61]. One reason for the low awareness of CPGs could be that the entry level for German physiotherapists is vocational school education. Furthermore, physiotherapists report limited access to guidelines [19] and often do not know where to find them [20]. We hypothesize that the physiotherapy management of persons with hip or knee osteoarthritis will most likely not be guideline adherent and therefore needs to be improved, which is the aim of this study.

There are numerous frameworks, models, and theories that provide guidance in developing strategies to facilitate guideline implementation [62]. However, many guideline implementation studies do not report explicit theory use or theory is not used consistently and transparently [26,63]. Choosing the most appropriate theory-driven approach is challenging as such models can be complex, that is, difficult for both researchers and health care practitioners to understand and operationalize, or lack comprehensiveness [64]. Although the TDF and BCW are comparatively new implementation frameworks, they have already been used in several studies to understand and promote the uptake of evidence in clinical practice [65-67], including in the field of physiotherapy [68]. For this research project, we decided to use the TDF as it has proven to be a comprehensive and accessible way to understand the barriers and facilitators to best practice in primary and secondary care in Western countries [64]. There is evidence that TDF-based interview schedules achieve a broader range of responses than when using a nontheoretical approach [69]. Therefore, the TDF is described as an “inclusive” rather than a “selective” approach [70]. The TDF works within the BCW framework as it provides a more detailed understanding of the behavior components of capability, opportunity, and motivation. The BCW provides a systematic and pragmatic approach to developing implementation interventions. It encourages intervention designers to comprehensively consider the full range of options and select those that are most promising for the context. In addition, it allows for a detailed specification of the content using agreed labels and definitions [28].

### Conclusions

The results from our project will provide valuable knowledge concerning the development process and effectiveness of an intervention to implement hip or knee osteoarthritis guidelines in physiotherapy practice. We aim to develop an implementation intervention that can also be transferred to other health conditions, with modifications, if necessary. We hypothesize that successful implementation will lead to increased guideline adherence in physiotherapists, which in turn will improve the quality of care.

## Abbreviations

ActiveA: Active with osteoArthritis
BCT: behavior change technique
BCW: Behavior Change Wheel
CONSORT: Consolidated Standards of Reporting Trials
CPG: clinical practice guideline
GLA:D: Good Life with osteoArthritis in Denmark
TDF: Theoretical Domains Framework

## Data Availability

The data sets generated during the research project will be available on the Open Science Framework: 10.17605/OSF.IO/A7P3W.
